# Dynamics of breast imaging research: A global scoping review and Sino-Australian comparison case study

**DOI:** 10.1371/journal.pone.0210256

**Published:** 2019-01-10

**Authors:** Seyedamir Tavakoli Taba, Patrick C. Brennan, Sarah Lewis

**Affiliations:** Medical Imaging Optimisation and Perception Group (MIOPeG), Faculty of Health Sciences, University of Sydney, Sydney, NSW, Australia; Institut Català de Paleoecologia Humana i Evolució Social (IPHES), SPAIN

## Abstract

This study presents a quantitative analysis of global breast imaging research over the last eight decades. A dedicated Sino-Australian case study via a social network analysis (SNA) is included as China and Australia have a recent rapidly increasing number of research partnerships and strategic education/economic connections. Bibliographic data was extracted via Scopus and analysed for the social network parameters of degree centrality, closeness centrality, betweenness centrality and multiple cohesion measures in order to explore research collaboration networks at the organisational level. Within the last three decades there has been a tremendous increase in the publication rate within the scientific domain of breast imaging research, however, there is a significant lag in the development of this research area in China compared with Australia. Breast imaging research in China is considerably more insular, with less international collaboration and reduced variation between collaborators than Australia. The impact of national breast screening programs and novel cancer technologies upon collaboration networks is discussed alongside the ability of networks paradigm to reveal both frailties in research connections and to highlight networking strategies.

## Introduction

Research collaborations facilitate the transfer of new knowledge in research processes, enhance the ability to use available resources cost-effectively and help in the translation of research efforts into practice [1]. Research organisations and funding bodies in many countries are promoting research collaborations particularly at multidisciplinary and multinational levels through various policy measures. More recently, quantitative and qualitative assessment of research collaborations and publications has increased significantly in different scientific fields. Particularly, bibliometric methods have been used to assess the effects of national research strategies on researchers’ publication practices and the effects of funding procedures on the performance of national research systems [2]. It has been argued that network dynamics can vary between different countries, particularly in terms of intra-regional and extra-regional collaborations, and that national research strategies and research publications can change the presence of a particular country in regional and international scientific arena [1].

Analysis of research collaborations and addressing strengths and weaknesses of research strategies in “breast imaging” is of particular significance. Breast cancer is the most commonly screened and diagnosed cancer via medical imaging and many countries have dedicated national programs for the early detection of breast cancer, primarily using mammography and sonography. Equally, medical imaging is also at the forefront of complex breast cancer and associated pathology cases, where newer imaging technologies such as Magnetic Resonance Imaging (MRI), Digital Breast Tomosynthesis (DBT) and Breast Computed Tomography (CT) are often employed to visualise occult cancers or women at high risk of misdiagnosis due to dense mammographic breast tissue, otherwise known as Breast Density (BD). There have been significant advances in digital imaging technology in recent decades, allowing cancer screening, diagnosis and research to flourish in supportive health environments.

Alongside a global quantitative comparison, a comparison of breast imaging research in China and Australia represents an important case study due to government policy, including economical and educational dynamics between the two countries. Australia is geographically the closest Westernised country to China and the Australian Government has identified Asia, and specifically China, as their focus for strategic development of collaboration in educational excellence in the 21^st^ century, with China being Australia’s largest trading partner [3]. Chinese migration to Australia has increased significantly in the last three decades, with migrants from China having doubled from 2006 to 2016 and now representing 2.2% of the Australian population [4]. Asian women, generally, have a higher composition percentage of fibro-glandular breast tissue, also known as breast density (BD) and high BD is a risk factor for breast cancer. Thus, as Chinese migration grows, and Australia draws closer to China economically, investigating the dynamics of social networks cancer research is valuable for health policy and collaboration.

In terms of health services and breast cancer statistics, Australian women have a 90% five-year survival rate from a breast cancer diagnosis (improved from 72% in 1980) and the incidence rate has become stable in recent years, with current incidence rates approximately 64 women in 100,000 [5]. In 2017, the estimated mortality rate from breast cancer was 6.5% in Australia [5]. Australia has had a free biannual national screening program in place since 1992, with a participation rate of approximately 55% [6]. By comparison, much less is known about breast cancer in China however reported incidence rate was 41 per 100,000 women in 2012 [7] and the incidence rate has risen by around 4% per year in the past [8]. This means that by 2023, China will have reached parity with Australia, having an incidence of 64 new cases per 100,000 population. The mortality rate of Chinese women is difficult to document due to the lack of national cancer coordination however the total mortality rate is close to 25% [8, 9]. China is without a nation-wide organised screening program or national breast cancer screening guidelines, and although there has been some focused activity on rural women’s health with free mammographic screening, participation is low [10].

In this study, we investigate the characterisation of international collaborations in breast imaging research since the very first publications in the field and explore countries and research institutions that have substantially influenced this evolution over time. This will be used as the basis for a more specific Sino-Australian comparison enquiry in which we examine the development of breast imaging research in China and Australia. We investigate the epidemiological research lines of breast imaging literature between the two countries as well as the research collaborations among different research institutions. The main objective of the current study is to identify the institutions that hold central or strategic positions in breast imaging collaboration networks, find out the differences in research networking in China and Australia, and examine these differences against research policies in breast imaging in the two countries.

Some studies have examined breast imaging research in the past, however, the number of such studies seems to be limited. One example of bibliometric analysis in this field is a two-page article by Ha, et al. [11] that quantitatively analysed global trends of breast cancer imaging research over 20 years from 1992 to 2012. The other study is by Tavakoli Taba, et al. [12] who focused their research on “mammography performance”, exploring how research publications in that area have evolved longitudinally over three decades (1984–2013). Glynn, et al. [13] also studied the broad area of “breast cancer” associated literature in terms of research quantity and quality. The study showed the ongoing expansion of breast cancer research and identified the key nations and journals involved in its’ development. The other work is an analysis of general “ultrasound” research by Chen, et al. [14] which focused on the most frequent topics of medical ultrasound research around the world. With the exception of one study [12], there was little or no quantitative examination of research connections in the aforementioned studies. The current paper will address this gap in the literature. The result from this study should be of high value for both researchers in the field of breast imaging and research policy makers at institutional and national levels as it may help to better understand the effects of research strategies upon publication practices and performance.

## Methods

Bibliographical data was extracted with the help of lexical search methods from Scopus, which is the largest international database of peer-reviewed literature. We searched for all publications that included one of the following phrases in the title: ‘breast imaging’, ‘mammogram’, ‘mammography’, ‘mammographic’, ‘breast ultrasound’, ‘breast ultrasonography’, ‘breast magnetic resonance imaging’, ‘breast MRI’, ‘breast computed tomography’, ‘breast CT’ or ‘breast tomosynthesis’. These words were chosen to represent the most common and important technological methods of imaging breast tissue in the last 30 years. The temporal range (publication year restriction) applied to the search was from the first available publication until 2017 (exclusive). Books and book chapters were excluded from the search, but no restriction was applied to the document language. After removing some duplications, the extracted dataset included 31,093 publications from 1937 to 2016. This large dataset was used to provide a holistic picture of breast imaging literature around the world.

Subsequently, we focused on two smaller cohorts of interest, being research related to either China or Australia. Among all extracted publications, firstly we retrieved the papers which had one of the words ‘China’, ‘Chinese’, ‘Hong Kong’ or ‘Macau’ in either the title, the abstract or the keywords. This resulted in 388 published papers which were related to China. We also filtered the publications with the words ‘Australia’ or ‘Australian’ in either the title, the abstract or the keywords. This time, we retrieved 307 publications which were related to Australia. Some data points (around 5%) in each dataset needed some data cleaning. This included missing data or spelling errors in country name or institution name of the affiliation data for some authors in the two datasets. Before being able to do any analysis based on the research collaboration networks, the affiliation data in the extracted datasets needed to be complete. We conducted manual data cleaning by identifying incomplete/incorrect data as much as possible and then replacing/modifying them with correct data with the help of information available on Google Scholar and institutional websites. The total number of publications in each dataset was not changed because of this data cleaning process.

Research collaborations can be studied as a set of nodes (social actors) and ties (relationships or links) between those nodes and social network analysis (SNA), as the process of investigating, mapping and measuring relationships between social structures, is commonly used to provide a deeper understanding of research collaborations [12, 15]. For the SNA in this study, if an author had more than one affiliation in a paper, only their first affiliation was used in the analysis. We used UCINET software [16] to analyse the networks.

### Social network measures

The centrality of a node refers to its position in the network. Bavelas [17] and Leavitt [18] were the first to practically employ the concept of centrality. Later, Freeman [19] contributed to this field by theoretically and experimentally expanding the intuitive concept of centrality and explaining centrality in terms of three major measures; “degree centrality”, “closeness centrality”, and “betweenness centrality”.

Degree centrality of a given node refers to the number of nodes adjacent/tied to that node. It is considered as an indicator to measure a node’s communication activity. Previous research references degree centrality as the extent to which a node can directly impart influence on other nodes (without any mediator) [20, 21]. The normalised degree centrality is the degree centrality divided by its maximum value in the network.

Closeness centrality is also defined as the extent of interconnectivity of a node with all other nodes in the network. Closeness reciprocates the “farness” which is the sum of the lengths of the shortest paths from a given node to every other node in the network. Newman [22] argues that closeness centrality determines the rapidity in the flow of information from one node to the others. The normalised closeness centrality of a given node is the reciprocal of its farness divided by the minimum possible farness in the network.

Betweenness centrality is the measure of the extent to which a given node occurs on the shortest paths between all pairs of node [23]. Betweenness centrality is considered as the power of a node to provide effective communication among other nodes in the network [19]. Borgatti [21] also refers to betweenness centrality as the capability of a node to obtain and control information in the network. The normalised betweenness centrality can be obtained by dividing betweenness centrality by the maximum possible value in the network.

While centrality measures can provide very useful information about the structural position of individual nodes in a network, they do not consider the broader structure of the network. As a result, “centralization” measures have been developed to determine the corresponding structural cohesion in an entire network. The centralization of a network shows the level of centrality of the most central nodes in the network in relation to all other nodes. Centralization of a network can be worked out by dividing the sum of variations in the centrality of individual nodes by the maximum centrality variation possible in the network [19]. For example, degree centralization of a network can be obtained by dividing the variation in the degree centrality of all nodes in the network by the maximum variation in the degree centrality scores in a network of the same size. This is also the case for closeness and betweenness centralization; however, closeness centralization is only measurable if there is a path between every node in the network.

In SNA, two nodes are members of the same “component” if there is a path connecting them. When analysing a network, we are also interested in the number and structure of the network components. This is mainly because the overall structure is not necessarily a reflection of the local structures in the network. For example, a network might be cohesive in local regions but not at the network global level, i.e. consider a network with high (average) degree centralities but with many split components. In such a network, information in each component flows easily from one node to the other but it is not transferable to other components.

Several measures have been developed to differentiate the cohesion at local and global levels. One measure is called “component ratio” and can be obtained by dividing the number of components by the number of nodes in the network. Higher component ratio is associated with more isolation in the network. The other measure is connectedness, which is defined as the proportion of pairs of nodes that can reach each other by any path [24]. In other words, connectedness is the proportion of pairs of nodes that are in the same component. The distance weighted measure of connectedness (where paths connecting nodes weighted inversely by their length) is called compactness [25]. In a compact network, every node is just a few steps far from any other. For both connectedness and compactness, the maximum value is one (when every node is adjacent to all others) and the minimum value is zero (when the network is completely made up of isolated nodes).

When studying research collaborations, the frequency of interactions between two nodes can be considered as the quantity of weighted tie or tie strength [26, 27]. However, to calculate most of the measures discussed above we needed to dichotomise the data matrix such that any tie strength larger than zero was recorded as one.

## Results and discussions

### Worldwide descriptive statistics

**Fig 1:**
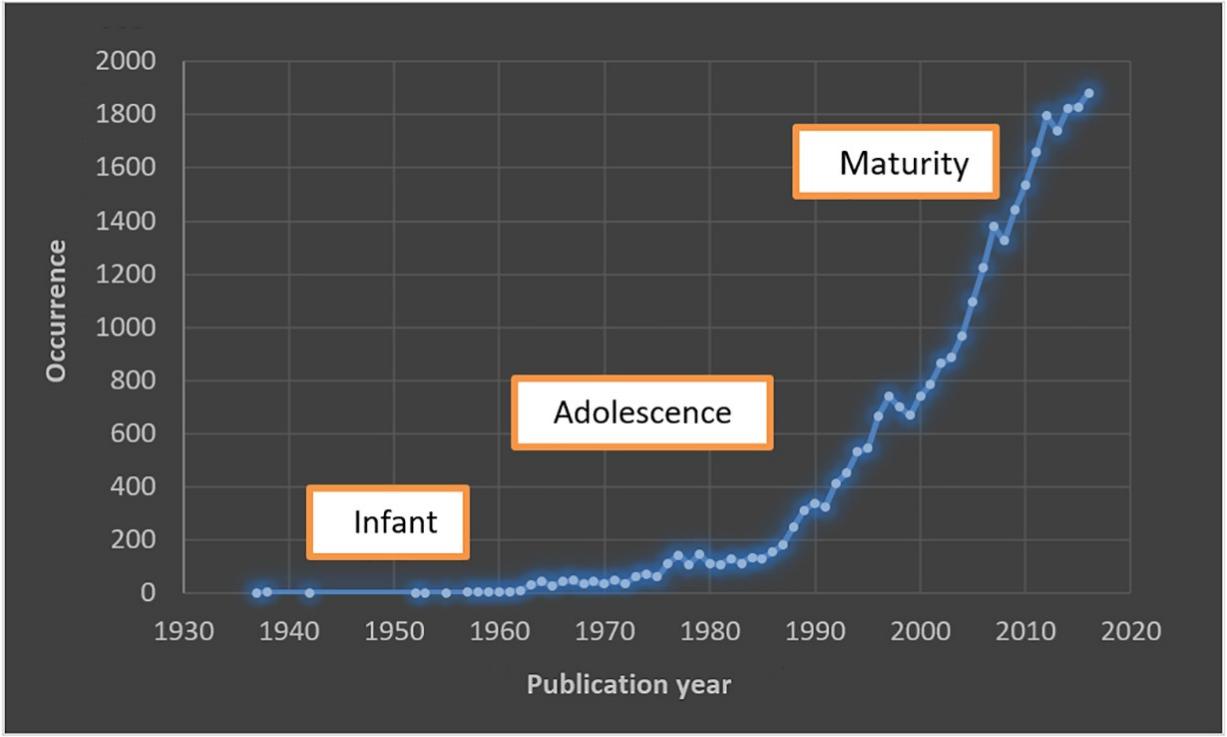
The world-wide evolution of breast imaging research.

Fig 1 shows the worldwide evolution of breast imaging research over 80 years from 1937 to 2016. The graph shows that the history of breast imaging publications can be regarded as three periods of approximately 25 to 30 years each. In the first period, from the late 1930s to the early 1960s, breast imaging can be considered an infant science and approximately 25 papers were published, mostly focusing on the feasibility of mammographic imaging using all-purpose (or general) x-ray machines in order to diagnose breast cancer during pre-surgery. The second period, which we shall call adolescence, started in the mid-1960s when actual mammography units as we know today were first introduced and in a time when breast Xerography emerged as a medical imaging tool [28]. This period continued through the 1970s when breast ultrasound was presented for the first time and the initial mammography screening trials were successfully conducted. By the late 1980s, mammography was emerging as standard practice for screening and diagnostic purposes in many developed countries and breast ultrasound was accepted as a breast imaging technique on its own account in Europe [29]. This was a start for the third period, called maturity, which has continued until now. Breast MRI began at the beginning of the maturity. Another important event in this period was the introduction of digital mammography in the early 2000s, which has gradually replaced film mammography over the last decade.

**Fig 2:**
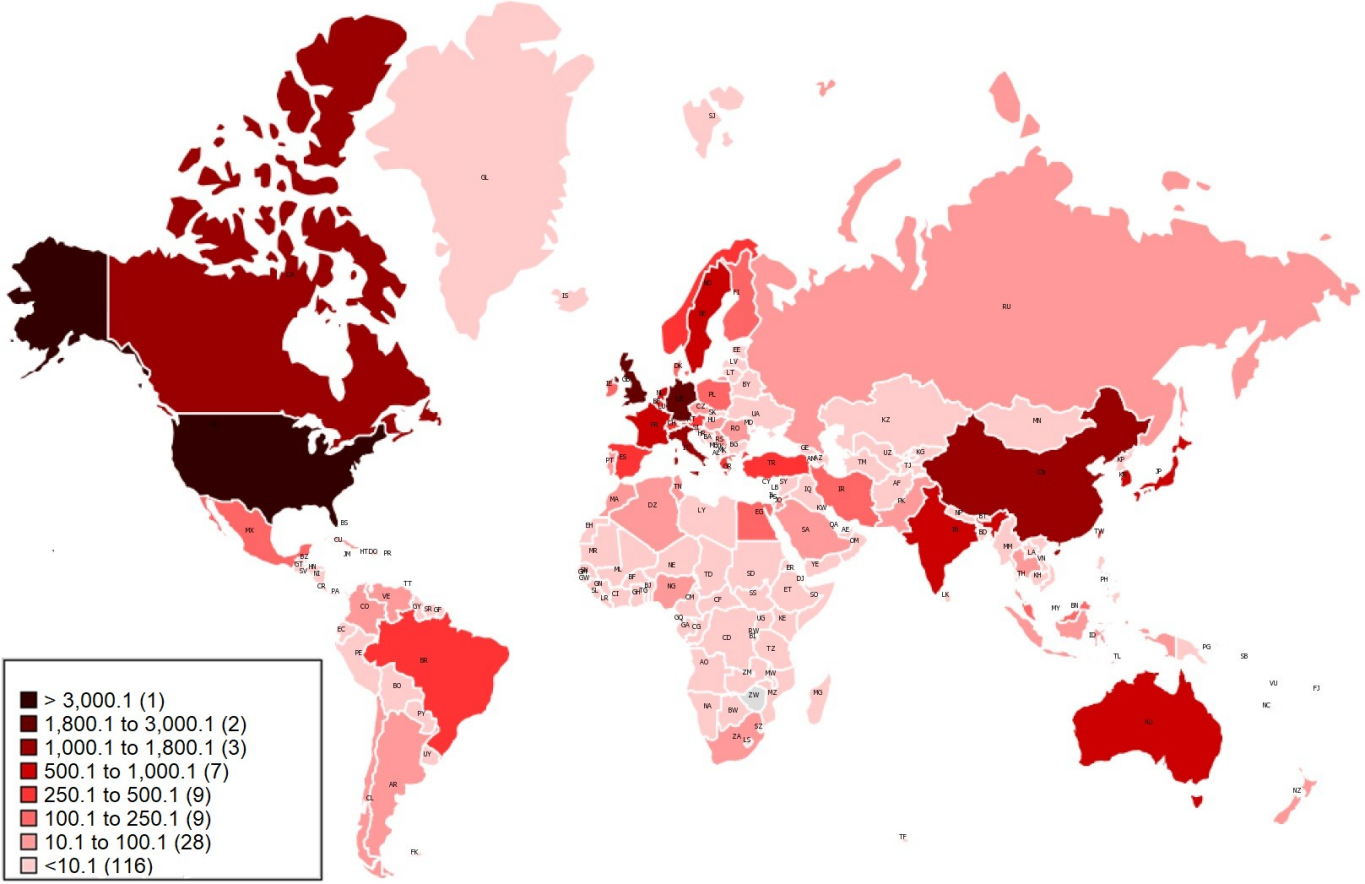
A map of countries (colour coded) in worldwide breast imaging research based on the frequency of each country’s affiliation in the dataset (1937–2016).

Fig 2 shows the frequency of breast imaging research by each country using author affiliation data from all breast imaging publications since 1937. The country “count” is not based on the relevance of the research to a particular country, but rather of times the affiliated nation occurs in the author affiliation dataset. Table 1 shows the top affiliation countries (with at least 500 counts) in breast imaging research and the rate of publication per million population, where population for each country was obtained from United Nations Department of Economic and Social Affairs [30].

**Table 1:** The affiliation countries with at least 500 publications in word-wide breast imaging research.

Ranking	Country	Count	Percentage of all counts	Population in million	Count per million of population
**1**	United States	11,075	35.8%	324.5	34.1
**2**	United Kingdom	1,966	6.4%	66.2	29.7
**3**	Germany	1,872	6.0%	82.1	22.8
**4**	China	1,574	5.1%	1,417.5	1.1
**5**	Italy	1,306	4.2%	59.4	22.0
**6**	Canada	1,294	4.2%	36.6	35.3
**7**	Japan	986	3.2%	127.5	7.7
**8**	France	924	3.0%	65.0	14.2
**9**	South Korea	880	2.8%	51.0	17.3
**10**	Netherlands	872	2.8%	17.0	51.2
**11**	Australia	787	2.5%	24.5	32.2
**12**	India	634	2.0%	1,339.2	0.5
**13**	Sweden	557	1.8%	9.9	56.2

The United States has been by far the most productive country in the domain with 11,075 counts, followed by the United Kingdom (1,966) and Germany (1,872). China (including Mainland China, Hong Kong and Macau) and Australia were both among the top 15 countries with 1,574 and 787 count, respectively. Sweden, Netherlands and Canada had the highest count of published breast imaging research per million population. Fig 2 concurs with Ha, et al. [11] in identifying the US as the most prolific research nation in the field of breast imaging, followed by the UK, although the Ha, et al. study only includes the period of 1992–2012.

The count of universities and research institutes in the author affiliation dataset also revealed the most world-widely productive organisations in breast imaging research. The top five organisations were University of California San Francisco (416), Harvard Medical School (405), University of Toronto (392), University of Texas MD Anderson Cancer Centre (359), and University of Washington Seattle (350), respectively.

### China-Australia descriptive statistics

**Fig 3:**
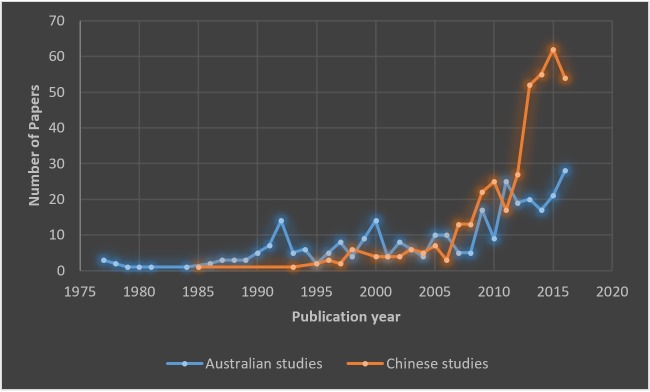
The evolution of breast imaging research in China and Australia.

We used a lexical search in the title, abstract and keywords of all extracted papers to source breast imaging studies which related to China and Australia. Fig 3 compares the development of breast imaging literature in China and Australia. The total number of breast imaging research papers relating to each country was similar with 388 studies for China and 307 for Australia. The first Chinese study in the area of breast imaging was published in 1985 and the second one in 1993, the maturity period, indicating a significant lag in breast imaging research despite a global awakening by China’s economy, international relations and population growth. Following 1993, the number of Chinese studies grew slowly for several years, with a subsequent amplification in the last several years, from 17 papers in 2011 to 62 in 2015 and 55 in 2016. The substantial increase in the number of Chinese breast imaging research in recent years could be a result of the focused rural screening program for women’s health in maternal/reproductive cancers. This campaign [31] was launched by the Ministry of Health of China and All-China Women's Federation between 2009 and 2015 in two phases to provide free cervical and breast cancer screening for millions of rural women, with ultrasound being the primary method for screening. On the other hand, the first retrieved Australian studies go back to 1977, within the adolescence period. After that, the number of Australian research publications in this domain has increased gradually from just a few papers per year in the 1980s to 28 papers in 2016.

**Fig 4:**
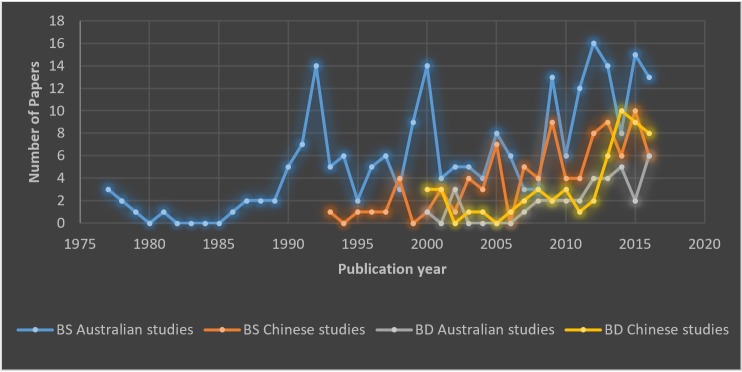
The epidemiological research lines of breast imaging literature in China and Australia; breast screening related (BS) and breast density related (BD).

As a sub-section of analysis, ‘breast density’ (BD) and ‘breast screening’ (BS) were included for the Sino-Australian comparison as these terms reflect two of the most common current epidemiological research lines and topical areas of interest for public health and research between the two countries. To do this, we conducted a lexical search of the words “density” and “screening” in the title, abstract and keywords of the Sino-Australian papers. Fig 4 displays the number of BD and BS papers over different years. The results show that 14.2% (55) of the Chinese studies and 11.1% (34) of the Australian studies were focussed on BD. For both countries, the first BD papers were published in 2000 reflecting a latent research period between the first quantification of BD in the early 1990s via the BIRADS system of mammographic reporting [32]. Since the early 2000s, the number of BD papers have increased gradually, almost with the same growth rate of the total breast imaging publications. On the other hand, the results show that while BS was 23.7% (92) of the Chinese studies, it was 73.3% (225) of the Australian studies, likely due the widespread acknowledgment of the federally funded BreastScreen Australia program, which partners with many universities and cancer-related government organisations. The peaks in Fig 4 are presumably related to some important national or international events in the history of breast imaging. For example, the first peak in 1992 is the year that Australian government introduced BreastScreen Australia as the national screening program for the early detection of breast cancer, and the second peak in 2000 is the year that the US Food and Drug Administration (FDA) approved the first digital mammography system. Increased number of studies in 2011–2012 could also be related to the FDA’s approval of 3D mammography technology, known as Digital Breast Tomosynthesis (DBT) in 2011.

### Publication diversity

With regards to publication sources, the data revealed that although there were a similar number of breast imaging publications related to each country, Chinese studies have been published in more diverse sources (journals and conference proceedings) compared with Australian research; we found 139 publication sources for the Chinese studies and 89 sources for the Australian studies. Table 2 shows the top 10 sources where breast imaging research related to China and Australia was published. This table also shows the impact factor and the number of related papers published in each journal. Journal citation reports impact factor [33] is often used in academia as a quantitative tool for ranking, evaluating, and comparing journals. Although this is not necessarily an indication of publication quality, it can be seen that Australian researchers are more inclined to publish their work in journals with an impact factor.

The top 10 sources, altogether, have published more than half of all publications related to each country, 52% of Chinese and 55% of Australian publications. The results indicated that the Chinese researchers tend to publish in their national sources: for the Chinese studies, 7 out of 10 top sources were considered national or local sources, whereas Australian researchers had only 4 of their top 10 sources as national sources.

**Table 2:** Top 10 publication sources for breast imaging research in China and Australia.

	Source Name	Publication Place	Impact factor	Number of papers	Portion of all papers
**Chinese studies**	Chinese Journal of Medical Imaging Technology	China	N/A	76	19.6%
	Chinese Journal of Radiology	China	N/A	57	14.7%
	Hong Kong Journal of Radiology	Hong Kong	N/A	16	4.1%
	National Medical Journal of China	China	N/A	12	3.1%
	Cancer Epidemiology Biomarkers and Prevention	United States	4.14	8	2.1%
	Asian Pacific Journal of Cancer Prevention	South Korea	2.51	7	1.8%
	Chinese Journal of Interventional Imaging and therapy	China	N/A	7	1.8%
	Hong Kong Medical Journal	Hong Kong	1.11	6	1.5%
	Journal of the Hong Kong College of Radiologists	Hong Kong	N/A	6	1.5%
	Cancer	United States	5.24	5	1.3%
**Australian studies**	Journal of Medical Imaging and Radiation Oncology	Australia	1.19	50	16.3%
	Medical Journal of Australia	Australia	2.87	43	14.0%
	Journal of Medical Screening	England	1.86	16	5.2%
	Radiography	England	N/A	13	4.2%
	Australian and New Zealand Journal of Public Health	Australia	1.69	10	3.3%
	Australian Journal of Public Health	Australia	Ceased	9	2.9%
	Asia Pacific Microwave Conference Proceedings	N/A	N/A	8	2.6%
	Breast	England	2.80	8	2.6%
	Breast Cancer Research and Treatment	United States	3.63	7	2.3%
	Cancer Epidemiology Biomarkers and Prevention	United States	4.14	6	2.0%

### Social network analysis

**Fig 5:**
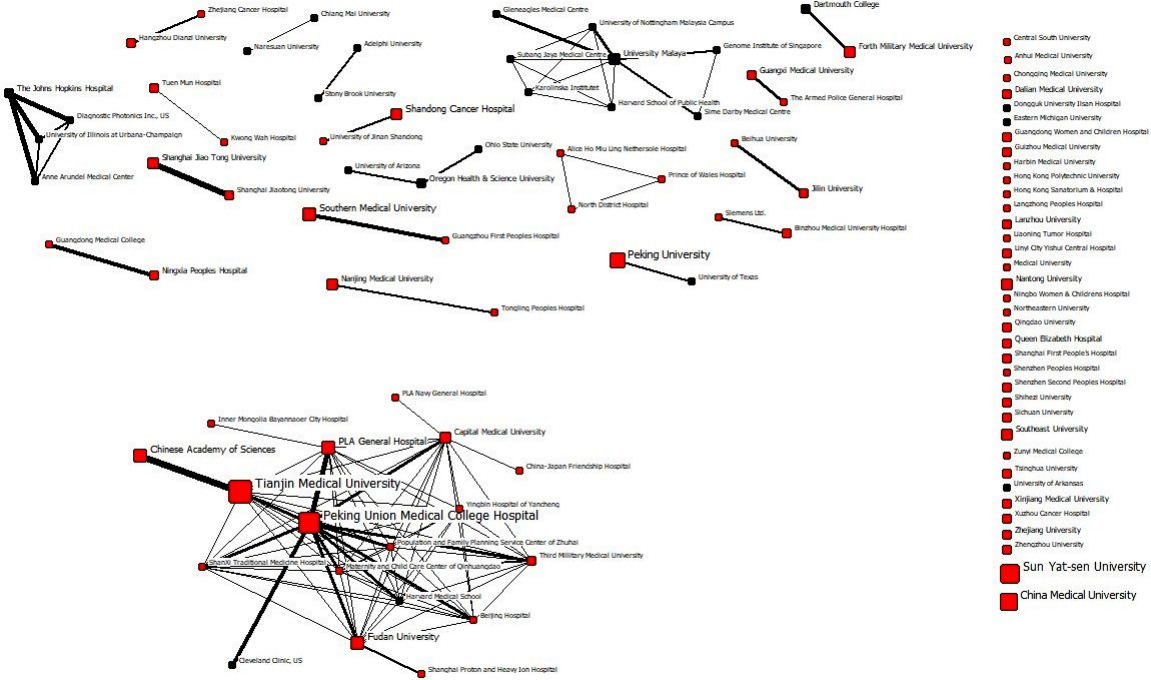
All-time research collaboration network of Chinese breast imaging research at the organisational level; the orange nodes denote Chinese research organisations and the black nodes represent international research organisations.

**Fig 6:**
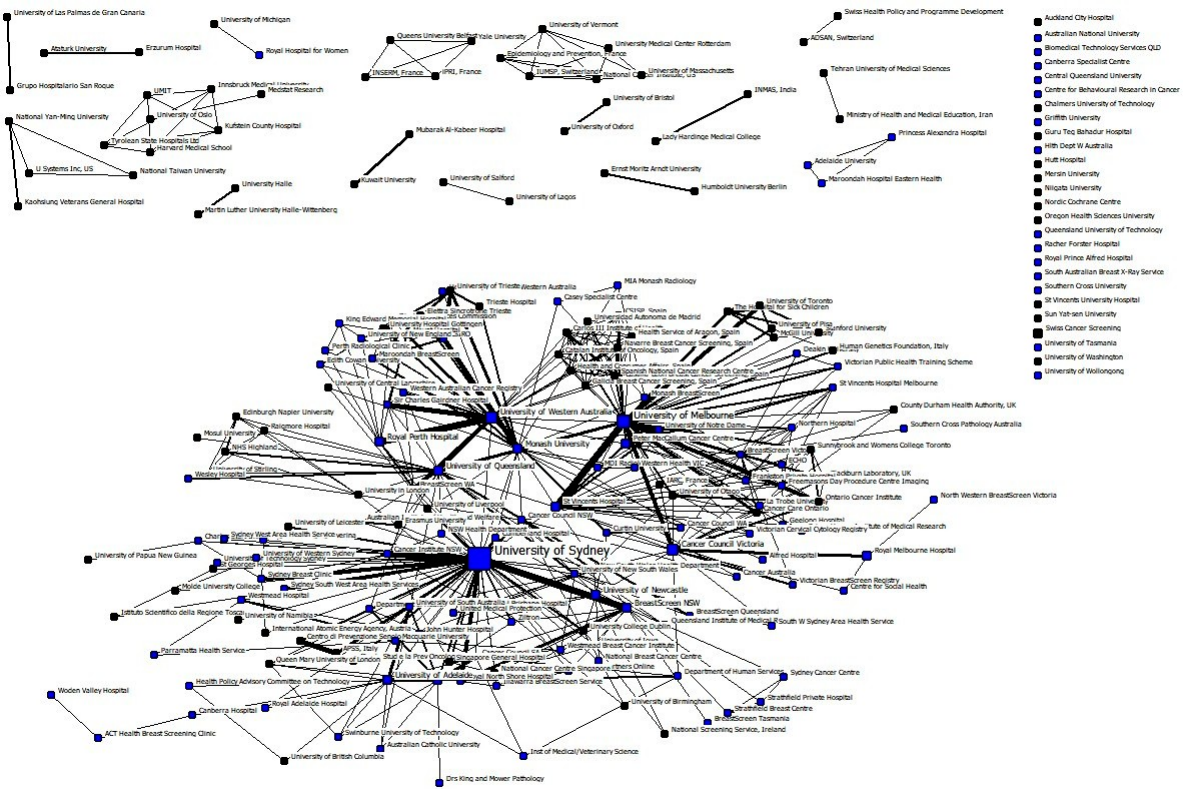
All-time research collaboration network of Australian breast imaging research at the organisational level, the blue nodes denote Australian research organisations and the black nodes represent international research organisations.

Fig 5 and Fig 6 shows the overall co-authorship relationships among research organisations in Chinese and Australian studies, respectively. In these sociograms, each node represents an organisation where the node size is associated with the count of papers published by that organisation. Likewise, each tie indicates the presence of a co-authorship relationship between two organisations where the tie width is associated with the frequency of interactions. In Chinese studies, 26% of organisations were non-Chinese while in Australian studies 48% were non-Australian. This substantial difference shows that the researchers in this domain have been more local in China compared with Australian researchers who have sought more international collaborations. Table 3 shows the highest productive organisations in Chinese breast imaging research and their count. Tianjin Medical University (54), Peking Union Medical College Hospital (50), Sun Yat-sen University (42), China Medical University (33) and Peking University (29) were the top research institutions in Chinese studies. For Australian studies, as shown in Table 4, University of Sydney (204), University of Melbourne (86), Cancer Council Victoria (51), University of Western Australia (50), and BreastScreen NSW (38) were the most influential nodes with regards to the count of publications.

**Table 3:** Top 10 affiliations in Chinese breast imaging studies and their normalised centrality measures.

Ranking	Organisation	Count	Degree Centrality	Closeness Centrality	Betweenness Centrality
**1**	Tianjin Medical University	54	0.121	0.012	0.003
**2**	Peking Union Medical College Hospital	50	0.121	0.012	0.003
**3**	Sun Yat-sen University	42	0	0.010	0
**4**	China Medical University	33	0	0.010	0
**5**	Peking University	29	0.010	0.010	0
**6**	PLA General Hospital	22	0.121	0.012	0.003
**7**	Southern Medical University	20	0.010	0.010	0
**8**	Fudan University	19	0.121	0.012	0.003
**9**	Chinese Academy of Sciences	19	0.010	0.012	0
**10**	Shandong Cancer Hospital	17	0.010	0.010	0

**Table 4:** Top 10 affiliations in Australian breast imaging studies and their normalised centrality measures.

Ranking	Organisation	Count	Degree Centrality	Closeness Centrality	Betweenness Centrality
**1**	University of Sydney	204	0.241	0.014	0.238
**2**	University of Melbourne	86	0.201	0.014	0.104
**3**	Cancer Council Victoria	51	0.085	0.014	0.039
**4**	University of Western Australia	50	0.121	0.014	0.076
**5**	BreastScreen NSW	38	0.067	0.014	0.029
**6**	Royal Perth Hospital	37	0.040	0.014	0.009
**7**	University of Newcastle	29	0.054	0.014	0.013
**8**	University of Queensland	26	0.098	0.014	0.069
**9**	University of Adelaide	23	0.058	0.014	0.037
**10**	Monash University	22	0.094	0.014	0.070

Table 3 and Table 4 show the normalised degree, closeness and betweenness centralities of each affiliation in Chinese and Australian studies. In the Chinese network, Capital Medical University had the highest centrality measures followed by Tianjin Medical University, Peking Union Medical College Hospital, PLA General Hospital, and Fudan University. Sun Yat-sen University and China Medical University, although having relatively high counts of papers, had no research collaboration in relation to breast imaging publications with any other organisations and so had zero centrality in the network. In the Australian network, University of Sydney was the most central organisation followed by University of Melbourne, University of Western Australia, University of Queensland, and Monash University. Overall, the normalised degree centrality and betweenness centrality measures of the highest productive organisations were considerably higher in the Australian network compared with the Chinse network. However, normalised closeness centralities were quite low in both networks as a result of the fact that many nodes were not connected to the main component. It is worth noting that in calculating closeness centralities, undefined distances between two nodes were replaced with the total number of nodes in the network (N).

Examining the entire networks, rather than individual nodes, also provide very useful information about research collaboration networks of Chinese and Australian studies. Table 5 illustrates some important entire network measures in the two countries. The overall number of collaborations (inter-organisational and intra-organisational) were substantially different in the two groups of studies: the average number of authors per paper in the Chinese studies was 1.77 compared with 4.06 in the Australian studies. For each country, this figure was calculated by counting the number of papers for each author, summing up the counts for all authors and dividing the result to the total number of papers.

**Table 5:** Entire network measures for Chinses and Australian breast imaging research.

Network measure	China	Australia
**Avg number of authors per paper**	1.77	4.06
**Portion of national affiliations**	74%	52%
**Portion of international affiliations**	26%	48%
**Avg Degree centrality (weighted ties)**	7.840	16.780
**Avg Degree centrality**	2.220	4.489
**Degree Centralization**	0.111	0.223
**Betweenness Centralization**	0.006	0.235
**Component Ratio**	0.545	0.192
**Connectedness**	0.042	0.463
**Compactness**	0.032	0.180

According to Table 5, both average degree centrality and average weighed degree centrality were smaller in the Chinese network than the Australian one. Also, the Chinese network had considerably smaller degree and betweenness centralizations, meaning that, overall, centrality was more uniform across different organisations in this network compared to the Australian network. Higher centralizations in the Australian network, on the other hand, show that there were certain organisations that behaved differently from the others in the network. These organisations (such as University of Sydney and University of Melbourne) hold central and strategic positions in the network and were playing a bridging role among otherwise disconnected organisations. These central organisations also helped to shape a big and relatively cohesive main component in the network (see Fig 6).

It should be noted that closeness centralization was not computable for either networks because of the presence of unconnected components. In this situation, component ratio, connectedness and compactness are more meaningful than closeness centralization. The Chinese network comprised one (relatively small) main component and 54 other smaller or isolated components while the Australian network consisted of one (relatively large) main component and 43 other smaller or isolated components. The component ratio was higher in the Chinese network showing less cohesion in this network compared with the Australian network. The proportion of pairs of nodes that had a direct way (of any distance) to each other, measured by connectedness, was also very small in the Chinese network (one-tenth of the same measure in the Australian network). Finally, the distance weighted connectedness, compactness, showed that, in average, nodes are farther from each other in the Chinese network, resulting in the consumption of more energy and time to transfer information to other nodes.

## Conclusion

The worldwide breast imaging literature has been constituted by over 31,000 journal articles and conference papers published between 1937 and 2016. Through a span of over 80 years of publication, the last 30 years can be considered as the maturity period of this scientific domain in which the publication rate increased tremendously. Almost 95% of all breast imaging papers have been published in this period, with more than 50% in the last 10 years. With regards to the number of publications, researchers from the United States have made the biggest contribution to breast imaging research (accounting for more than one-third of all counts) followed by the United Kingdom and Germany. The University of California San Francisco, Harvard Medical School and University of Toronto have been the global research pioneers in this domain.

Examining breast imaging research related to China and Australia as two case studies with an overall similar number of publications (388 and 307 papers, respectively), we found a significant lag in the development of this research area in China compared with Australia. This could be mainly the result of limited federal financial support for breast screening research in China, as opposed to the symbiotic relationship between BreastScreen Australia and universities, together with the associated impact upon public awareness that comes from national screening programs. While the BreastScreen Australia program was established in 1992 and provided a valuable fabric for collaboration between many universities and research organisations to conduct breast imaging research, the first national breast screening campaign in China was launched just in 2009 and has been limited to rural locations [10]. Indeed, Wang, et al. [10] suggest there is an awakening of breast cancer research as related to the sharp increase in prevalence and the shift towards private, paid screening by the Chinese middle class, especially on the Eastern seaboard of China. The results from this study showed that breast imaging research in China has been more local, or insular, when compared with Australia which has attracted or sought international collaborations. For example, there were substantially lower numbers of international collaborators in the Chinese research collaboration network and there was a tendency for Chinese researchers to publish their research in national sources rather than seek international peer review. National and institutional research strategies in China might need to further encourage Chinese researchers to develop international collaborations in order to facilitate rapid knowledge transfer in the area of breast cancer imaging and invite external evaluation of research and practise processes.

The research collaboration networks of the two countries were quite different based on various social network metrics. The average authors per paper in the Chinese research was around half of the Australian counterpart. Average degree centrality, as a measure of inter-organisational connectedness and direct influence, was also much lower in the Chinese network. Degree and betweenness centralizations were larger in the Australian network, showing more variations in the centrality measures of different nodes and the significant role of the most central nodes in this network. The University of Sydney and University of Melbourne had the highest count and the highest centrality measures in the Australian network. Such higher education institutions connected otherwise disconnected research centres and shaped a large cohesive main component in the Australian network. In the Chinese network, where there were fewer variations among different nodes, Tianjin Medical University and Peking Union Medical College Hospital had the highest count, but they were very much less central compared with their Australian counterparts. Overall, all network cohesion measures, including component ratio, connectedness and compactness, showed that the Chinse network was less cohesive than the Australian network, meaning the slower flow of new information and knowledge in the Chinese network. Again, a nation-wide organised screening program or directive, along with offering multi-institutional and multidisciplinary grant opportunities, would help Chinese organisations to develop a more effective breast cancer imaging network.

The data presented in this paper should be of value to health policy makers, researchers and academic strategists, specifically in China and Australia but also beyond, where the interactions between key collaborators of medical imaging research are clearly highlighted. In particular, this study shows how national and institutional research strategies, as well as national health programs (such as BreastScreen Australia), may affect publication practices and research collaborations in a country. Furthermore, this paper indicates that scientific breakthroughs in areas of cancer risk, for example, a new understanding related to breast density, combined with public interest has enormous implications for research dissemination and future health policy direction.

This study was limited to a general scoping review and a SNA case study, but future studies can investigate the associations between network measures and scientific impact (for example citations) in the medical imaging research community. The ability of the social networks paradigm to reveal frailties in research connections, highlights where networking strategies are needed. Such strategies should ultimately enable a better and more comprehensive understanding of the impact of health policy change around population-based screening and the lasting value of novel cancer technologies.

## Supporting information

S1 File PRISMA checklist. (PDF)
